# The Difference in Serum Metabolomic Profiles between the Good and Poor Outcome Groups at 3 Months in the Early and Late Phases of Aneurysmal Subarachnoid Hemorrhage

**DOI:** 10.3390/ijms25126597

**Published:** 2024-06-15

**Authors:** Brigitta Orban, Roland Tengölics, Laszlo Zavori, Diana Simon, Szabina Erdo-Bonyar, Tihamer Molnar, Attila Schwarcz, Peter Csecsei

**Affiliations:** 1 Department of Neurosurgery, Medical School, University of Pecs, 7632 Pecs, Hungary; orbanbrigi8@gmail.com (B.O.); schwarcz.attila@pte.hu (A.S.); 2 Metabolomics Lab, Biological Research Centre, Hungarian Research Network, 6726 Szeged, Hungary; tengolics.roland@brc.hu; 3 Core Facilities, Biological Research Centre, Hungarian Research Network, 6726 Szeged, Hungary; 4 Hungarian Centre of Excellence for Molecular Medicine—Biological Research Centre Metabolic Systems Biology Lab, 6726 Szeged, Hungary; 5 Emergency Department, Saudi German Hospital, Dubai 391093, United Arab Emirates; zavori.laszlo@gmail.com; 6 Department of Immunology and Biotechnology, Medical School, University of Pecs, 7632 Pecs, Hungary; simon.diana@pte.hu (D.S.); erdo-bonyar.szabina@pte.hu (S.E.-B.); 7 Department of Anaesthesiology and Intensive Care, Medical School, University of Pecs, 7632 Pecs, Hungary; molnar.tihamer@pte.hu

**Keywords:** aneurysmal subarachnoid hemorrhage, metabolomics, outcome, energy metabolism

## Abstract

We aimed to investigate the characteristics of serum metabolomics in aneurysmal subarachnoid hemorrhage patients (aSAH) with different 3-month outcomes (good = modified Rankin score: 0–3 vs. poor = mRS 4–6). We collected serum samples from 46 aSAH patients at 24 (D1) and 168 (D7) hours after injury for analysis by liquid chromatography-mass spectrometry. Ninety-six different metabolites were identified. Groups were compared using multivariate (orthogonal partial least squares discriminant analysis), univariate, and receiving operator characteristic (ROC) methods. We observed a marked decrease in serum homocysteine levels at the late phase (D7) compared to the early phase (D1). At both D1 and D7, mannose and sorbose levels were notably higher, alongside elevated levels of kynurenine (D1) and increased 2-hydroxybutyrate, methyl-galactoside, creatine, xanthosine, p-hydroxyphenylacetate, N-acetylalanine, and N-acetylmethionine (all D7) in the poor outcome group. Conversely, levels of guanidinoacetate (D7) and several amino acids (both D1 and D7) were significantly lower in patients with poor outcomes. Our results indicate significant changes in energy metabolism, shifting towards ketosis and alternative energy sources, both in the early and late phases, even with adequate enteral nutrition, particularly in patients with poor outcomes. The early activation of the kynurenine pathway may also play a role in this process.

## 1. Introduction

The subarachnoid hemorrhage caused by aneurysmal rupture (aSAH) can potentially be fatal or lead to severe disability. The overall incidence of aSAH is approximately 9 per 100,000 person–years [1]. Patients with aSAH had a short-term mortality rate of 18.4% and a 5-year mortality rate of 29%. At the 5-year follow-up, 64.0% of patients were alive and free from disabilities [2]. The most common primary causes of neurological devastation are direct effects of the primary hemorrhage (55%), aneurysm rebleeding (17%), and medical complications (15%) [3]. Well-established risk factors for unfavorable outcomes and mortality include poor clinical grade at presentation, older age, aneurysm rebleeding, large aneurysm size, and cerebral infarction from vasospasm [3]. Early brain injury (EBI) is increasingly recognized as a critical determinant of mortality and disability following aSAH, driven by various pathophysiological processes such as neuroinflammation, oxidative stress, brain tissue hypoxia, and blood–brain barrier disruption [4]. Recent studies also point to post-ischemic mitochondrial dysfunction [4] and disturbed cerebral energy metabolism as significant factors in the pathology of aSAH patients [5]. Significant global alterations in cerebral energy metabolism in all cerebrovascular territories independently of aneurysm location were observed in aSAH patients [6]. Similar disturbances of the cerebral energy metabolism were found after traumatic brain injury [7]. Another study with severe aSAH patients indicates that cerebral energy dysfunction is frequent and is associated with normal to hyperemic CBF [8]. Overall, based on literary data, it is likely that significant changes affecting energy metabolism occur during the early brain injury (EBI) following subarachnoid hemorrhage. However, the dynamics, precise direction, and long-term impact of these changes on prognosis remain largely unclear. 

In the present study, we aim to identify metabolomic features in early and late serum samples obtained from aSAH patients with different prognoses using an ultrahigh-performance liquid chromatography–tandem mass spectroscopy (UPLC–MS/MS) system. These insights could help clinicians better understand the pathophysiological and metabolic changes occurring during aSAH.

## 2. Results

### 2.1. Patients’ Characteristics

We analyzed serum samples obtained from aSAH patients (n = 46) with good outcomes (n = 16) and poor outcomes (n = 30) using untargeted LC–MS metabolomics. The two outcome groups were age- and gender-matched (*p* > 0.05). The distribution of mRS scores was as follows: mRS 0: 1 (2%), mRS 1: 11 (24%), mRS 2: 4 (8.7%), mRS 3: 7 (15%), mRS 4: 11 (24%), mRS 5: 8 (17%), mRS 6: 4 (8.7%). Compared with the good outcome group, patients with poor outcomes had higher mFisher and WFNS scores, and more patients required mechanical ventilation and extraventricular drainage (all *p* < 0.001). The admission CRP (*p* = 0.027), lymphocyte count (*p* = 0.023), and serum glucose level (*p* = 0.028) were significantly higher in the group with poor 3-month outcomes compared to the favorable outcome group. Similarly, there were significantly more infections (*p* = 0.001) and patients requiring nasogastric enteral feeding in the poor outcome group (*p* < 0.001). Detailed characteristics of patient groups are shown in Table 1.

### 2.2. Comparison of Serum Metabolomic Profiles According to Early and Late Sampling Times in aSAH Patients

A total of 92 serum metabolites were detected. The metabolite quality data can be viewed in the Supplementary Material (Table S1). Serum metabolomic changes between D1 and D7 were explored by comparing the metabolomic profiles from different sample time points with OPLS-DA (cumulative Q2:0.505, R2Y: 0.777), Suppl. Figure S1A. T-test and fold change (FC) analysis were used to screen differential metabolites. The criteria for screening differential metabolites were VIP (variable importance in projection) > 1.0, Log2 FC ≥ 1.2 or <0.8333, and *p*-value <0.05. A total of 14 metabolites were identified, of which 4 were up-regulated, and 10 were down-regulated (Table 2). Volcano plots and VIP values are displayed in Suppl. Figure S1B,C. Only homocysteine had a high enough AUC value (AUC = 0.838), which signifies good discriminative ability between early and late sampling time points.

### 2.3. Serum Metabolic Profiles of aSAH Patients with Good and Poor Outcome

**Figure 1:**
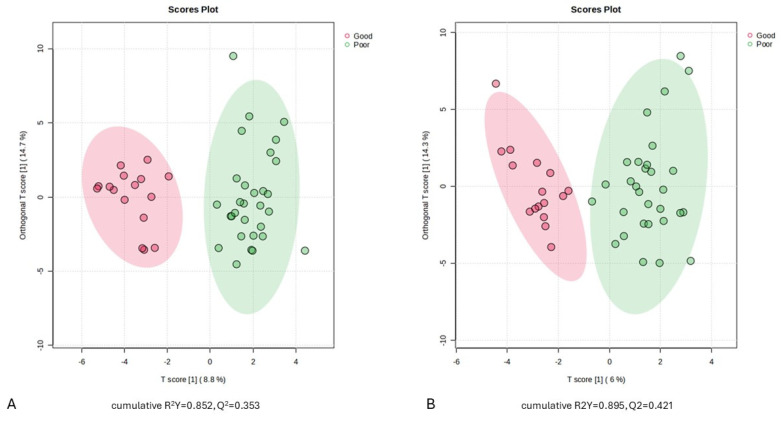
The score plots of the OPLS-DA models: (**A**) (D1), (**B**) (D7). The horizontal axis in the figure is the first principal component, and the vertical axis is the second principal component. The number in parenthesis is the score of that principal component, which indicates the percentage of the overall variance explained by the corresponding principal component. D1, sampling time 24 h after injury; D7, sampling time 168 h after injury.

To accurately identify the differential metabolites in the serum of aSAH patients with good and poor outcomes, the samples were further analyzed using the OPLS-DA model at both time points (D1 and D7). The OPLS-DA score plots showed moderate discrimination between the metabolomic profiles of the good and poor outcome groups at the D1 sampling time point (Figure 1A, cumulative R^2^Y = 0.852, Q^2^ = 0.353). Slightly better discrimination is observed when applying the model between the two groups at the D7 sampling time point (Figure 1B, cumulative R2Y = 0.895, Q2 = 0.421). 

### 2.4. Screening of Differential Metabolites in aSAH Patients between Outcome Groups

T-test and fold change analysis (FC analysis) were used to screen differential metabolites. The criteria for screening differential metabolites were VIP (variable importance in projection) ≥ 1, Log2 FC ≥ 1.2 or < 0.8333, and *p*-value < 0.05; they are summarized in Table 3. Based on these criteria, a total of 5 differential metabolites were identified on D1, and 19 differential metabolites were identified on D7, of which 2 (D1) and 9 (D7) were up-regulated, and 3 (D1) and 10 (D7) were down-regulated. 

**Figure 2:**
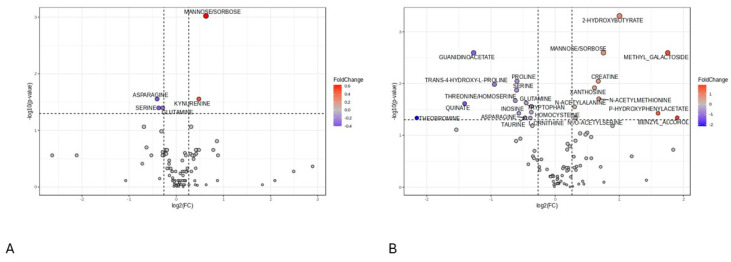
Differential metabolites screening. Volcano plots: (**A**) (D1) and (**B**) (D7). Red represents up-regulated metabolites, and blue represents down-regulated metabolites. Grey represents neither up- or down-regulated metabolites. D1, sampling time 24 h after injury; D7, sampling time 168 h after injury.

The unfavorable outcome was characterized by an increase in mannose/sorbose and kynurenine and a decrease in asparagine, serine, and glutamine on day 1 after injury (D1). On day 7 (D7) after injury, there was an increase in 2-hydroxibutyrate, methyl-galactoside, mannose/sorbose, creatine, xanthosine, N-acetil-methionine, P-hydroxiphenylacetat, benzyl-alcohol, and N-acetylalanine in patients with poor outcome vs. the patients with good outcome at 3 months. We detected a decrease in the following metabolites in patients with a poor prognosis on D7: guanidinoacetate, proline, serine, trans-4-hydroxi-l-proline, threonine, glutamine, quinate, tryptophan, inosine, homocysteine. The intensity difference in metabolites discriminating between the two groups at D1 and D7 time points is depicted in Supplementary figures (Figure S2) as box plots. Volcano plots show differences in these metabolites between the good and poor outcome groups on D1 and D7 (Figure 2A,B).

### 2.5. Analysis of Differential Serum Metabolites and Metabolic Pathways

**Figure 3:**
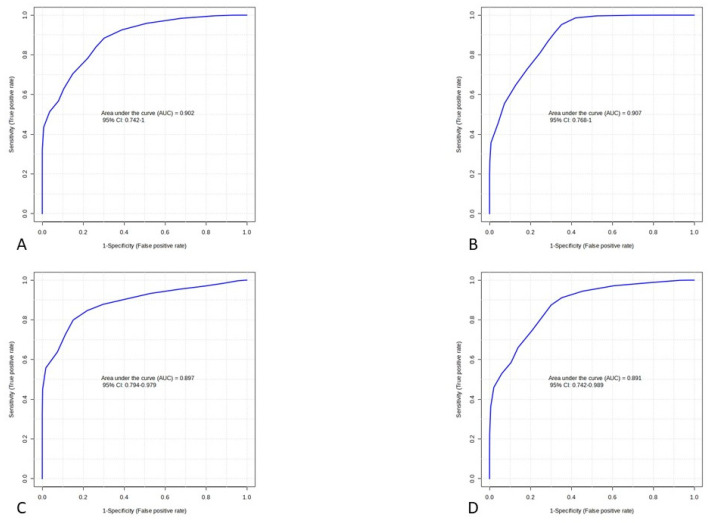
Power of the biomarker panel to distinguish between good and poor 3-month outcome groups: (**A**) clinical markers on D1, (**B**) metabolomic markers on D1, (**C**) clinical markers on D7, (**D**) metabolomic markers on D7. AUC, area under the curve, CI, confidence interval. The clinical markers were found to be independent predictors (age, WHNS) in binary logistic regression analysis, and their combined predicted probabilities were used. D1, sampling time 24 h after injury; D7, sampling time 168 h after injury.

A receiver operating characteristic (ROC) curves were generated to evaluate the predictive value of differential metabolites for poor outcomes in aSAH patients. Figure S3 in Supplementary figures shows the ROC curves of those metabolites whose AUC value is >0.8 (good accuracy). Metabolomic markers that met the criteria for differential metabolites (VIP ≥ 1, Log2 FC ≥ 1.2 or <0.8333, and *p*-value < 0.05) outperformed clinical biomarkers in predicting poor outcomes at an early sampling time point (D1), as shown in Figure 3. Among the D7 metabolites, only those with a *p*-value < 0.001 were included in the model. Subsequently, the various metabolic pathways were analyzed using MetaboAnalyst 5.0.

**Figure 4:**
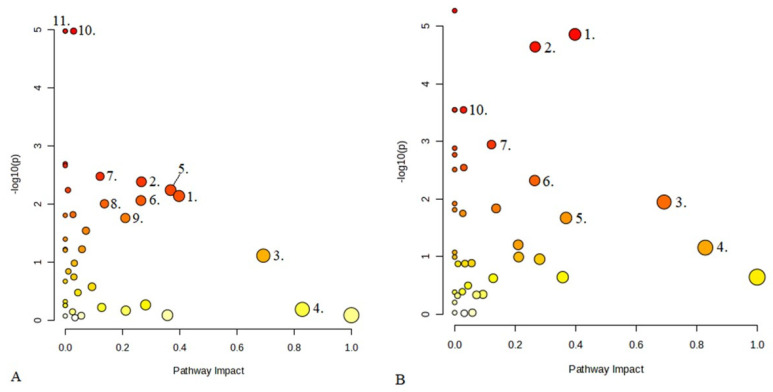
Metabolic pathway analysis: (**A**) D1; (**B**) D7. The horizontal coordinate indicates the pathway impact value, the vertical coordinate indicates the −log10(*p*) of the pathway, a dot in the figure represents a metabolic pathway, the size of the dot is proportional to its impact value, and the color of the dot represents the size of the pathway *p*-value, where the color change from yellow to red represents the change in the *p*-value from large to small. Metabolic pathways: 1. arginine and proline metabolism; 2. glycine, serine, and threonine metabolism; 3. caffeine metabolism; 4. taurine/hypotaurine metabolism; 5. tryptophan metabolism; 6. cysteine and methionine metabolism; 7. glioxylate and dicarboxylate metabolism; 8. arginine metabolism; 9. alanine metabolism, asparagine, and glutamate metabolism; 10. galactose metabolism; 11. amino sugar and nucleotide sugar metabolism.

We selected clinical markers for outcome prediction based on binary logistic regression analysis. On univariate analysis, age, admission CRP, mFisher score, and WFNS showed associations with the outcome, binary logistic regression identified age (OR: 1.112, 95% CI:1.019–1.214, *p* = 0.017), and WFNS (OR:3.462, 95%CI:1.238–9.513, *p* = 0.018) as independent predictors of the outcome. Therefore, we used their combined predictive probability in the clinical predictive model. The general overview of metabolic pathways in D1 and D7 is shown in Figure 4.

## 3. Discussion

In this study, we utilized the UPLC-MS/MS system to examine the changes in the metabolomic profile of aSAH patients between day 1 and day 7 post-aSAH, as well as differences between groups with good and poor 3-month outcomes. The most important findings can be summarized as follows:

(1) At 24 h post-injury, the group with poor 3-month outcomes exhibited significantly elevated levels of mannose/sorbose and kynurenine alongside reduced levels of amino acids.
(2) At 168 h following the injury, significantly higher levels of 2-hydroxybutyrate, methyl galactoside, mannose/sorbose, creatine, xanthosine, N-acetylmethionine, N-acetylalanine, and p-hydroxyphenylacetate were detected in the serum of patients with poor 3-month outcomes.
(3) At 168 h post-aSAH, the poor outcome group exhibited significantly lower levels of guanidinoacetate. Moreover, certain essential amino acids were also found at reduced levels during both the early and late sampling times in this group. Conversely, concentrations of specific N-acetyl amino acid derivatives were elevated in the poor prognosis group at the late sampling time.
(4) Between the two sampling times, homocysteine showed significantly lower serum levels in the late samples compared to the early ones.

After 24 h following initial SAH, based on metabolomic measurements, we observed a significant increase in kynurenine levels in the group with poor outcomes. Kynurenine serves as a sensitive marker of neuroinflammation in the acute phase of neurological disorders associated with inflammation [9]. Previous research has shown that activation of the kynurenine pathway is associated with poor outcomes in critically ill patients [10], as well as with higher mortality and larger infarct volumes in early-phase ischemic stroke patients [11]. Additionally, it is significantly overactivated during inflammation in traumatic brain injury [12]. These prior findings are consistent with those observed in the current study. During the acute phase, patients with aSAH (aneurysmal subarachnoid hemorrhage) and poor outcomes exhibit heightened inflammatory responses [13]. In a metabolomic study conducted on critically ill patients, higher levels of kynurenine, sucrose, and p-hydroxyphenylacetate showed an association with higher mortality rates [14]. The elevated kynurenine levels observed in the poor outcome group in this study underscore the presence of significant acute neuroinflammation. 

Guanidinoacetate (GAA) is a naturally occurring amino acid derivative that serves as a direct precursor of creatine [15] and is produced from arginine and glycine through the catalytic action of l-arginine: glycine amidinotransferase (AGAT), primarily in the kidneys and liver. Later, it is converted into creatine and homocysteine, predominantly by guanidinoacetate N-methyltransferase (GAMT), mainly in the liver but also in the brain. Accumulation of GAA in the brain and body fluids (serum levels > 16 μmol/L) is associated with muscle weakness, epilepsy, and intellectual disability in individuals with GAMT deficiency [16]. Conversely, GAA depletion (serum levels < 1.9 μmol/L) has been observed in patients with chronic kidney disease and diabetes mellitus [17]. GAA may act as an agonist for gamma-amino butyric acid (GABA)-A receptors and could potentially modulate GABA metabolism in the brain and peripheral tissues [18]. It also exhibits a vasodilatory effect [19] and can function as a phosphocreatine mimetic and an alternative energy donor, particularly when creatine availability is limited [20]. Its vasodilatory effect is achieved through two mechanisms: inhibiting norepinephrine methylation, thus affecting its half-life and sympathetic activity [21], and indirectly inducing vasodilation by sparing arginine and promoting arginine-mediated nitric oxide (NO) production [22].

In our current study, we found that on the 7th-day post-SAH, serum samples from the group with poor outcomes showed GAA depletion, higher creatine levels, and decreased homocysteine levels compared to the group with good outcomes. The lower GAA levels in the poor prognosis group could be attributed to impaired GAA production or increased utilization. One possible explanation for GAA depletion is the enhanced conversion to creatine, while the conversion to homocysteine is less pronounced. The decrease in available GAA levels could also be attributed to liver and kidney dysfunction resulting from severe conditions, which are prevalent in a significant portion of patients with severe conditions and poorer outcomes [23]. The low level of guanidinoacetate definitely draws attention to severe energy metabolism disturbance in the late stage of SAH. Furthermore, the lower GAA levels observed in the group with poor prognosis theoretically limit its vasodilatory and GABA agonist effects. However, the clinical significance of this observation remains uncertain and warrants further investigation.

In our study, significantly higher levels of 2-hydroxybutyrate were observed in serum samples taken on day 7 in the group with poor 3-month outcomes. During brain ischemia, the liver produces β-hydroxybutyrate, which is then consumed by the brain [24]. Clinical studies have demonstrated a linear correlation between the arterial concentration of ketone bodies (KB) and cerebral KB uptake [25,26]. In human studies, elevated KB values can appear as early as 72 h after the injury, depending on the degree of starvation [27]. 2-hydroxybutyrate is a ketone body that accurately predicts diabetic ketoacidosis in children and adolescents [28], and ketosis may represent an intermediate state of metabolic dysregulation rather than being associated with a more severe acute illness [29]. However, high levels of ketone bodies have numerous favorable effects on central nervous system functions. 2-hydroxybutyrate has been shown to improve neurological function in focal ischemia [30], reduce outcomes of global ischemia and stroke [31], and prevent neuronal death in models of Alzheimer’s and Parkinson’s disease [32,33]. It exerts multiple activities in the brain, including interaction with ion channels, inhibition of histone deacetylation [34], indirect antioxidative activity [35], and inhibition of neuroinflammation [36].

The production of β-hydroxybutyrate during brain ischemia may offer advantages as it can serve as an energy source for the brain, bypassing the need for glucose and reducing the risk of harmful lactate formation. β-hydroxybutyrate also reduces the peri-infarct glucose-metabolism-driven production of reactive oxygen species and astrogliosis, leading to improved neurogliovascular and functional recovery after ischemic insult [37]. BHB serves as an alternative carbon source, fueling oxidative phosphorylation (OXPHOS) and the production of bioenergetic amino acids and glutathione, which are crucial for maintaining redox balance [38]. Additionally, BHB supports T cell responses during infections and may indicate impaired ketogenesis.

Ultimately, elevated levels of 2-hydroxybutyrate may signal metabolic dysregulation due to acute subarachnoid hemorrhage. Alternatively, this increase could represent a compensatory response, indicative of a shift in energy production towards ketosis, which offers several beneficial effects on nervous system function.

In our study cohort, all patients received adequate enteral nutrition in accordance with clinical guidelines regarding both quantity and quality. The primary objective of enteral feeding in critically ill patients is to provide sufficient nutrition and support to satisfy their metabolic requirements and either maintain or enhance their nutritional status. In the group with poor outcomes, the higher levels of ketone bodies observed in the late phase are likely not a consequence of inadequate enteral nutrition but rather attributable to changes in energy metabolism independent from nutritional circumstances.

Overall, the presence of significantly higher levels of 2-hydroxybutyrate in serum samples taken on day 7 in the group with poor 3-month outcomes might suggest alterations in metabolic processes, potentially reflecting changes in energy metabolism or metabolic stress in individuals with adverse outcomes.

In our current study, we observed significantly lower amino acid levels in the poor outcome group compared to the good outcome group at both sampling time points, except for the concentrations of N-acetylmethionine and N-acetylalanine. Sjöberg et al. observed an increase in amino acid levels in the blood during the first week following subarachnoid hemorrhage [39] and described the moderate predictive power of serum myoinositol for a one-year favorable outcome. In a study involving 29 patients, amino acid levels were higher in the poor outcome group compared to the favorable outcome group, but the significance of the difference was very slight and was only observed in the early stage (0–3 days) [40]. In another study where plasma samples of aSAH patients were examined [41], no differences were found in the levels of amino acids between early and late samples, nor between the plasma amino acid levels of good and poor outcome groups; in fact, certain amino acid levels decreased in the plasma of aSAH patients compared to controls. Tuoho et al. observed a hypermetabolic state in patients with ICH and aSAH undergoing surgery [42]; similar results were observed in another study [43], additionally demonstrating that increased amino acid infusion had no effect on amino acid exchange.

Based on the literature data and our study, it could be concluded that the changes in amino acid metabolism detectable in the blood following aSAH are complexly influenced by numerous factors (severity, postoperative nutrition, etc.). 

During critical illness, the metabolism of macronutrients is altered at multiple levels. The utilization of energy substrates predominantly relies on the mobilization of endogenous stores, which is governed by a range of regulatory mechanisms [44]. The oxidation of carbohydrates is globally more increased during the early phase than the oxidation of lipids and proteins in critically ill patients [45]. Moreover, when increasing nitrogen intake while maintaining calorie load or changing only the composition of nitrogen carriers, the supply of essential amino acids could become inadequate [45]. Our results support this previous observation, as we found significantly increased mannose/sorbose levels and decreased amino acid levels in patients with poor outcomes, both in the early and late stages of aSAH.

Our study has several limitations. Due to the relatively low number of cases, the generalizability of the conclusions is limited. The pathophysiological processes occurring during SAH involve numerous metabolic and signaling pathways that were not investigated in the present study. Serum was used for the analysis, while simultaneous examination of cerebrospinal fluid (CSF) could have provided additional valuable insights. We did not collect daily data regarding the nutritional status of the patients; we calculated the daily energy requirement only after admission to the ICU.

In summary, the higher levels of 2-hydroxybutyrate and lower levels of guanidinoacetate and amino acids observed in the late phase in the group with poor outcomes following SAH may indicate a shift towards ketosis in energy metabolism, even if SAH patients receive adequate enteral nutrition during intensive care. Based on our findings, these biomarkers demonstrate potential suitability as indicators of disease severity in routine clinical care. Their implementation could facilitate immediate adjustments in both the quantitative and qualitative aspects of nutritional management. Moreover, leveraging the established protective role of ketosis may confer additional clinical benefits. Nevertheless, further research is warranted to substantiate these claims. Although the observed metabolic differences are likely not specific to subarachnoid hemorrhage, the detailed pathophysiological role of ketosis in the late phase of SAH should be subject to further investigation. 

## 4. Materials and Methods

### 4.1. Subjects and Study Design

Institutional review board approval was obtained previously (IV/8468-1/2021/EKU), and written informed consent was obtained from each patient or their legal representative. Inclusion criteria were age > 18 years, spontaneous aneurysmal SAH diagnosed by computed tomography (CT) within 24 h of ictus, and aneurysm detected on digital subtraction angiography (DSA). Exclusion criteria were: traumatic SAH, pregnancy, hospital admission later than 24 h after ictus, no aneurysm treatment, bleeding from arteriovenous malformation, absence of a signed consent form, underlying SARS-CoV-2 infection, and systemic diseases (tumors, liver and/or renal insufficiency, and chronic lung disease). Following the diagnosis of aSAH, according to our hospital standards, the aneurysm was treated endovascularly within 24 h. In all instances, the patients were admitted to the neurointensive care unit for a minimum duration of 12–14 days, allowing for timely detection of anticipated complications, such as symptomatic vasospasm. If symptomatic macrovascular vasospasm occurred based on the deterioration of the clinical status (the occurrence of focal neurological impairment or a decrease of at least 2 points on the Glasgow Coma Scale) [45], MR and MR angiography were performed to confirm macrovascular vasospasm on large vessels (ICA, MCA M1-M2, ACA, VA, BA) or DWI lesions. If the MR was not informative or was not clear, we performed a DSA and, if necessary, administered intra-arterial nimodipine. We applied early enteral nutrition either orally or via nasogastric tube, ensuring adequate daily energy intake.

### 4.2. Clinical Definitions Data Collections and Outcome Measures

Clinical data collected included age, sex, medical and social history (hypertension, diabetes, smoking), and neurological status at admission based on the World Federation of Neurosurgical Societies scale (WFNS grade) from each patient. Each CT scan was assessed independently by a neurologist involved in the study, who evaluated the extent and distribution of blood based on the modified Fisher score (mFS) categorization. Functional outcome was assessed using the 0–6 modified Rankin Scale (mRS). A poor outcome at three months post-discharge was defined as a mRS score of 3–6, categorizing this cohort as the poor outcome group. Conversely, individuals with an mRS score of 0–2 were classified as the good outcome group. The 3-month mRS was obtained via a follow-up phone call by a trained research fellow using a standardized questionnaire or through a personal interview during the control DSA examination. Subjects were treated according to standard guidelines [46,47]. We also recorded data on nutrition (oral, nasogastric tube, parenteral) and metabolic status (serum glucose and lactate) daily during the stay in the intensive care unit. All patients received nimodipine 6 × 60 mg per os from the first day for vasospasm prevention. Symptomatic vasospasm (SVP) was characterized by the narrowing of the arterial vessel lumen as observed in MR or cerebral angiography, accompanied by any new focal neurological sign or a decrease of at least two points on the Glasgow Coma Scale (GCS). The presence of SVP was assessed by visual inspection of cerebral angiography or source images of cerebral angiography. The diagnosis had to be confirmed by an independent observer (neuroradiologist). The definition criteria of systemic infection were as follows: symptoms of infection with fever, elevated C-reactive protein and/or procalcitonin, and a positive diagnostic test such as chest X-ray or urine test. The nutrition of patients included in the study was conducted according to the current guidelines for the nutrition of patients in the intensive care unit [46]. The energy requirement was calculated using the Harris–Benedict equation, which is also recommended for critically ill patients [46,48].

### 4.3. Sample Collection, Processing Protocol, and LC/MS Analysis

Serum samples were collected at four predetermined time points after ictus: 24 h (D1) and 168 h (D7). Arterial blood samples were collected in 9 mL plain glass blood tubes (BD Vacutainer ©) spun down, fractionated, and frozen in 2 mL polypropylene vials (Sarstedt AG & Co., Nümbrecht, Germany) at −80 °C within 30 min. The samples were stored at −80 °C until measurements.

Blood serum was thawed in ice. For LC/MS analysis, 400 µL acetonitrile was mixed with 100 µL serum samples in a 1.5 mL microcentrifuge tube. The mixture was vortexed for 5 s. This was followed by centrifugation at 20,000× *g*, at 4 °C, for 10 min, and the supernatant was collected. 

For metabolome analysis, an ultrahigh-performance liquid chromatography–tandem mass spectroscopy (UPLC–MS/MS) system, namely a Thermo Q-Exactive Focus instrument (Thermo Fisher Scientific, Waltham, MA, USA) equipped with a Dionex Unimate 3000 UHPLC system was used. For chromatographical separation, an Xbidge Premier BEH Amide column (2.1 mm × 150 mm, 2.5 μm; Waters Corporation, Milford, Massachusetts, USA) was used. From each sample, 5 µL was injected into the system. The mobile phase composition was gradually changed from 98% solvent A (97.5/2.5 acetonitrile/water to 80% solvent B (20/80 acetonitrile/20 mM ammonium acetate + 20 mM ammonia in water) in 9 min using a flow of 400 μL/min with gradient profile 6. After 1 min equilibration, the eluent composition returned to the initial conditions in 0.5 min, and the system was equilibrated for 4 min. For acquiring semi-quantitative metabolome data, the system was operated in MS1 scan mode using negative ionization. Data were acquired using the following settings: sheath gas: 55 l/min; aux gas: 14 l/min; sweep gas: 4 l/min; probe heater 440 °C; capillary temperature 280 °C with 2500 V capillary voltage in negative ionization mode. The scanning mass range was 100 to 800 m/z with an AGC target of 3 × 10^6^ and a maximum IT time of 250 ms. Pooled extract samples as QCs were injected multiple times across the batch to follow the guidelines of Broadhurst et al. [49]. For acquiring MS2 data, 10, 30, and 50 NCE were used for DDMS confirmation mode in both positive and negative ionization modes. 

### 4.4. Data Processing and Statistical Analysis

For metabolite identification and metabolite signal extraction exact masses (considering deprotonation only), the retention times of the MSMSL metabolite library were used. For obtaining peak intensity values, the recorded data were processed using Tracefinder software 5.1. Metabolite signal intensities (peak area) were extracted based on their retention time (+/− 0.25 min) and exact mass data (+/− 2.5 ppm). Metabolite data were normalized using PQN normalization and QC-RLSC normalization [50].

Typical metabolites found in serum sample selected for MS2 level confirmation (see Table S1) Compound Discoverer 3.2 was used for MS2 level putative metabolite identification, searching against the Endogenous Metabolites compound class of mzCloud database and in-house MS2 library on MSMLS metabolite library. Therefore, metabolites were identified at the highest available level [51], marked in Supplementary Table S1.

A web-based analytical software, Metaboanalyst 5.0, was used for the multivariate statistical analyses of metabolomics data, including the orthogonal partial least squares discriminant analysis (OPLS-DA), biomarker analyses, and pathway analyses. Benjamini–Hochberg false discovery rate (FDR) adjustment for Student’s t-test was performed on the MetaboAnalyst. OPLS-DA was conducted to visualize the possible global metabolic difference of patients between good and poor outcomes on D1 and D7. To validate the OPLS-DA model, different permutation tests were performed to evaluate OPLS-DA model reliability (n = 100), and R2Y and Q2 were utilized to assess its quality. According to the OPLS-DA analysis, the potentially different metabolites were selected by variable importance in the projection (VIP) values (VIP > 1). Additionally, the potential metabolic signatures with false discovery rate (FDR) ≤ 0.05 and fold change (FC) > 1.2 or <0.8333 were identified. Biomarker analysis was used to obtain the receiver operating characteristic (ROC) curve-based approach for identifying the potential biomarkers and evaluating their performance. The area under the curve (AUC) was generated to verify whether combining the identified metabolites could serve as a biomarker to distinguish between good and poor outcome groups. To identify clinical variables that could distinguish between good and poor outcomes, univariate and adjusted multivariate logistic regression analyses were performed. The differential metabolic pathways were analyzed by MetaboAnalyst 5.0 (https://www.metaboanalyst.ca, accessed 10 May 2024). Compared with the KEGG database, KEGG value > 0.5 and *p*-value < 0.05 were used as screening conditions for screening. Pathway analysis was employed to identify the various key biological pathways linked to the observed changes in metabolites.

## Figures and Tables

**Table 1:** Patients characteristics.

Variable	Good Outcome (N = 16)	Poor Outcome (N = 30)	*p*-Value
Age, years, mean ± SD	55 ± 10	57 ± 13	0.852
Female, N (%)	16 (70)	19 (83)	0.300
Hypertension, N (%)	9 (39)	4 (17)	0.077
NIDDM, N (%)	0 (0)	3 (13)	0.073
COPD, N (%)	1 (4)	1 (4)	1.000
IHD, N (%)	9 (39)	14 (61)	0.140
Current smoking, N (%)	14 (61)	11 (48)	0.375
Aneurysm localization, N (%)			
internal carotid	0 (0)	2 (9)	
middle cerebral artery	7 (30)	7 (30)	
a. comm. anterior	5 (22)	5 (22)	
a. comm. posterior	4 (18)	1 (4)	
a. cerebri anterior	1 (4)	1 (4)	
vertebrobasilar	6 (26)	7 (30)	
mFisher score, median (IQR)	2 (1–3)	3 (3–4)	<0.001
WFNS, median (IQR)	1 (1–2)	4 (2–5)	<0.001
Creatinine *, µmol/L, median (IQR)	56 (49–70)	60 (49–71)	0.700
CRP *, mg/L, median (IQR)	5 (2–15)	27 (2–73)	0.027
WBC *, G/L, median (IQR)	11 (8–12)	10 (9–12)	0.910
Neutrophile *, G/L, median (IQR)	7 (6–10)	8 (7–10)	0.381
Lymphocyte *, G/L, median (IQR)	2 (1–2)	1 (1–2)	0.023
Extraventricular drainage, N (%)	2 (9)	19 (83)	<0.001
Lumbal drainage, N (%)	11 (48)	12 (52)	0.768
Decompressive craniotomy, N (%)	0 (0)	2 (9)	0.148
Mechanical ventilation, N (%)	2 (9)	19 (83)	<0.001
Infection, N (%)	3 (13)	15 (65)	0.001
Symptomatic vasospasm, N (%)	3 (13)	7 (30)	0.153
Parenteral nutrition	0 (0)	0 (0)	1.000
Initiation of EN ^a^, hours, mean ± SD	33.5 ± 2.5	32.7 ± 3.5	0.465
Enteral nutrition (via NG tube)	4 (25)	24 (80)	<0.001
Enteral nutrition (oral diet)	15 (94)	9 (30)	<0.001
MRI ischemia, N (%)	2 (9)	5 (22)	0.218
Glucose ^b^, µmol/L, median (IQR)	7 (6.4–7.2)	7.3 (7–7.7)	0.028
Lactate ^b^, µmol/L, median (IQR)	0.8 (0.6–1)	0.9 (0.7–1.2)	0.195
3-month mRS, median, IQR	1 (1–3)	5 (4–5)	<0.001

Values are expressed as numbers (% of total), mean ± SD or median (IQR). SAH, subarachnoid hemorrhage; N, number; NIDDM, non-insulin dependent diabetes mellitus; WFNS score, World Federation of Neurosurgical Societies score; COPD, chronic obstructive pulmonary diease; IHD, ischemic heart disease; IQR, interquartile range; CRP, C-reactive protein; MRI, magnetic resonance imaging; NG, nasogastric tube; EN, enteral nutrition, * on admission, ^a^ mean time from ICU admission to initiation of EN, ^b^ The median (IQR) value of levels measured from the first (D1) to the 7th day (D7). Differences between groups were determined using the Kruskal–Wallis test with Bonferroni correction or Chi-squared test.

**Table 2:** List of differential metabolites between D1 and D7 in aSAH patients.

Metabolites	*p*-Value ^a^	FDR ^b^	FC ^c^	VIP ^d^	AUC
Homocysteine	<0.0001	0.0000	0.54299	2.5649	0.838
Pantothenate	<0.0001	0.0022	0.72975	2.0931	0.736
Theobromine	<0.0001	0.0022	0.27842	2.3236	0.732
Homogentisate	<0.0001	0.0022	1.4076	1.3739	0.736
Pregnenolone Sulfate	<0.0001	0.0023	0.27842	2.1133	0.727
Paraxanthine	0.0005	0.0054	0.16176	2.1269	0.706
Glycocholate	0.0006	0.0054	1.9743	1.7525	0.670
Methyl Galactoside	0.0014	0.0116	0.62255	1.8354	0.691
Quinate	0.0019	0.0149	0.72407	1.6022	0.685
Biliverdin	0.0032	0.0218	0.75389	1.7025	0.677
Xanthurenate	0.0042	0.0257	1.4583	1.6431	0.672
Glycochenodeoxycholate	0.0046	0.0267	2.0485	1.3549	0.670
Bilirubin	0.0051	0.0278	0.74985	1.6738	0.668
Glutamate	0.0071	0.0361	0.72695	1.4018	0.662

^a^ The *p*-values were calculated using two-tailed Student’s *t*-tests. ^b^ FDR was calculated using the Benjamini–Hochberg method. Metabolites with FDR values ≤ 0.05 were considered as significant. ^c^ Fold change was calculated based on the arithmetic mean value of each group. Metabolites were obtained when the Log2 FC ≥ 1.2 or < 0.8333. ^d^ Variable importance in the projection (VIP) was obtained based on OPLS-DA with a value > 1.0.

**Table 3:** List of differential metabolites between good and poor outcomes at sampling times D1 and D7 in aSAH patients.

Metabolites	*p*-Value ^a^	FDR ^b^	FC ^c^	VIP ^d^	AUC
D1					
Mannose/Sorbose	<0.0001	0.0009	1.5782	2.2981	0.868
Kynurenine	0.0006	0.0278	1.3946	2.2316	0.820
Asparagine	0.0009	0.0278	0.7551	1.8846	0.800
Serine	0.0020	0.0400	0.7720	1.7817	0.758
Glutamine	0.0022	0.0400	0.8195	1.7818	0.768
D7					
2-Hydroxybutyrate	<0.0001	0.0014	2.0034	2.2096	0.856
Methyl_Galactoside	0.0002	0.0050	3.3747	2.0167	0.820
Guanidinoacetate	<0.0001	0.0000	0.4144	2.0029	0.917
Mannose/Sorbose	0.0001	0.0031	1.6853	1.9738	0.835
Creatine	0.0004	0.0063	1.5934	1.8054	0.808
Proline	0.0003	0.0062	0.6618	1.7326	0.812
Serine	0.0015	0.0142	0.6596	1.6987	0.779
Trans-4-Hydroxy-L-Proline	0.0012	0.0121	0.5188	1.6620	0.785
Threonine/Homoserine	0.0067	0.0327	0.6503	1.6316	0.741
Xanthosine	0.0022	0.0183	1.5301	1.6144	0.770
Glutamine	0.0046	0.0282	0.7317	1.5320	0.752
N-Acetylmethionine	0.0006	0.0073	1.6020	1.4859	0.802
Quinate	0.0042	0.0279	0.3762	1.4790	0.754
P-Hydroxyphenylacetate	0.0039	0.0277	3.0389	1.4328	0.756
Benzyl_Alcohol	0.0067	0.0327	3.7385	1.3918	0.741
Tryptophan	0.0010	0.0113	0.7737	1.3769	0.789
Inosine	0.0105	0.0481	0.6753	1.3634	0.729
N-Acetylalanine	0.0031	0.0235	1.2340	1.3633	0.762
Homocysteine	0.0063	0.0327	0.7728	1.3288	0.743

^a^ The *p*-values were calculated using two-tailed Student’s *t*-tests. ^b^ FDR was calculated using the Benjamini–Hochberg method. Metabolites with FDR values ≤ 0.05 were considered as significant. ^c^ Fold change was calculated based on the arithmetic mean value of each group. Metabolites were obtained when the Log_2_ FC ≥ 1.2 or < 0.8333. ^d^ Variable importance in the projection (VIP) was obtained based on OPLS-DA with a value > 1.0. D1, sampling time 24 h after injury; D7, sampling time 168 h after injury; FDR, false discovery rate; FC, fold change; AUC, area under the curve.

## Data Availability

Data supporting the findings of this study are available upon reasonable request, and detailed metabolite data will be uploaded to a Zenodo public repository.

## Supplementary Materials

The supporting information can be downloaded at: https://www.mdpi.com/article/10.3390/ijms25126597/s1.
