# Supplementary material for: The Difference in Serum Metabolomic Profiles between the Good and Poor Outcome Groups at 3 Months in the Early and Late Phases of Aneurysmal Subarachnoid Hemorrhage
Source: Int J Mol Sci. 2024 Jun 15;25(12):6597. doi: 10.3390/ijms25126597 (PMC11203497; doi:10.3390/ijms25126597)
Supplement: Supplementary file 1 [file ijms-25-06597-s001.zip › Legend Table S1.pdf]

metabolite_name: Name of the metabolite based on MSMLS library of IROA tech: https://www.iroatech.com/mass-spectrometry-metabolite-library-of-standards-msmls/

retention_time: Retention time of the given compound

Blank_cnt: number of blank samples, which contained peak at the given compound specific retention time and exact mass.

blank_average_intensity: average peak intensity in blank samples

QC_average_intensity: average peak intensity in pooled extract sample

QC_cv: relative standard deviation of the given metabolite peak intensity in pooled extract samples

na_proportion_of_biological_sample: proportion of biological samples in which the given metabolite peak wasn't found

MS2_verified: the given metabolite identification verified with fragmentation and mzCloud - https://www.mzcloud.org/ - library match also

Kegg_ID: metabolite ID in kegg database

HMDB_ID: metabolite ID in HMDB database
