# Supplementary material for: The Difference in Serum Metabolomic Profiles between the Good and Poor Outcome Groups at 3 Months in the Early and Late Phases of Aneurysmal Subarachnoid Hemorrhage
Source: Int J Mol Sci. 2024 Jun 15;25(12):6597. doi: 10.3390/ijms25126597 (PMC11203497; doi:10.3390/ijms25126597)
Supplement: Supplementary file 1 [file ijms-25-06597-s001.zip › Supplementary Figures.pdf]

The Difference in Serum Metabolomic Profiles between the Good and Poor Outcome Groups at 3 Months in the Early and Late Phases of Aneurysmal Subarachnoid Hemorrhage

Brigitta Orban [1], Roland Tengölics [2,3,4], Laszlo Zavori [5], Diana Simon [6], Szabina Erdo-Bonyar [6], Tihamer Molnar [7], Attila Schwarcz [1] and Peter Csecsei [1,*]

1 Department of Neurosurgery, Medical School, University of Pecs, 7632 Pecs, Hungary; orbanbrigi8@gmail.com (B.O.); schwarcz.attila@pte.hu (A.S.)
2 Metabolomics Lab, Biological Research Centre, Hungarian Research Network, 6726 Szeged, Hungary; tengolics.roland@brc.hu
3 Core Facilities, Biological Research Centre, Hungarian Research Network, 6726 Szeged, Hungary
4 Hungarian Centre of Excellence for Molecular Medicine—Biological Research Centre Metabolic Systems Biology Lab, 6726 Szeged, Hungary
5 Emergency Department, Saudi German Hospital, Dubai 391093, United Arab Emirates; zavori.laszlo@gmail.com
6 Department of Immunology and Biotechnology, Medical School, University of Pecs, 7632 Pecs, Hungary; simon.diana@pte.hu (D.S.); erdo-bonyar.szabina@pte.hu (S.E.-B.)
7 Department of Anaesthesiology and Intensive Care, Medical School, University of Pecs, 7632 Pecs, Hungary; molnar.tihamer@pte.hu
* Correspondence: csecsei.peter@pte.hu

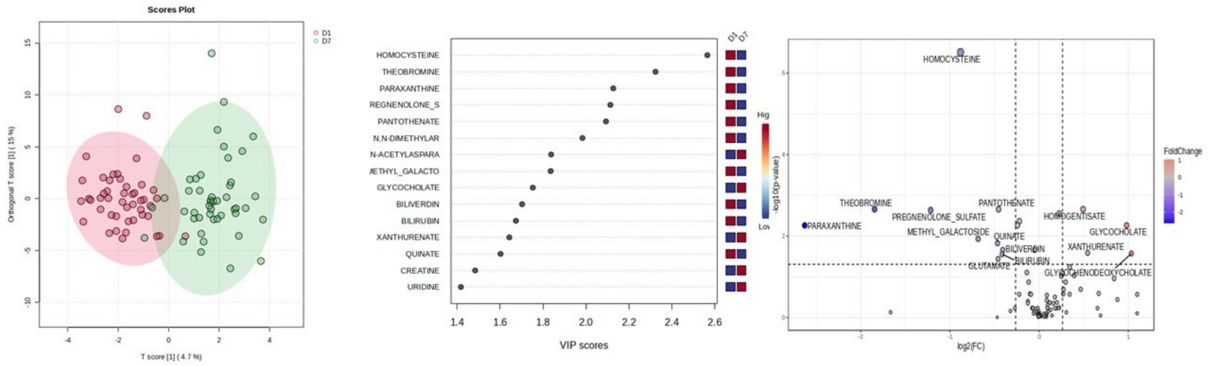

A **B** **C**

Figure S1. (A) The score plot of the OPLS-DA model. The score plots of the OPLS-DA models. The horizontal axis in the figure is the first principal component, and the vertical axis is the second principal component, The number in parenthesis is the score of that principal component, which indicates the percentage of the overall variance explained by corresponding principal component. (B) VIP value score graph. The vertical coordinate in the graph indicates a metabolite, and the horizontal coordinate indicates

the VIP value. (C) Volcano plots. Red represents up-regulated metabolites, and blue represents down-regulated metabolites. Grey represents meaningless metabolites.

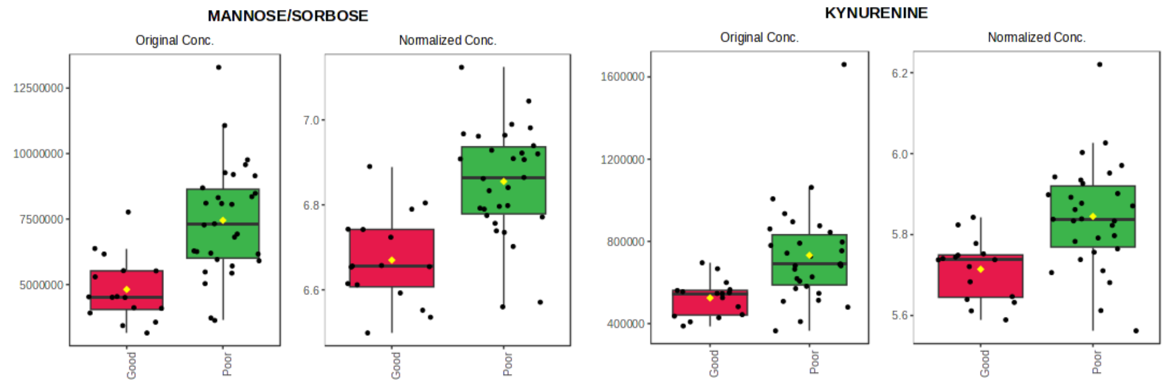

A

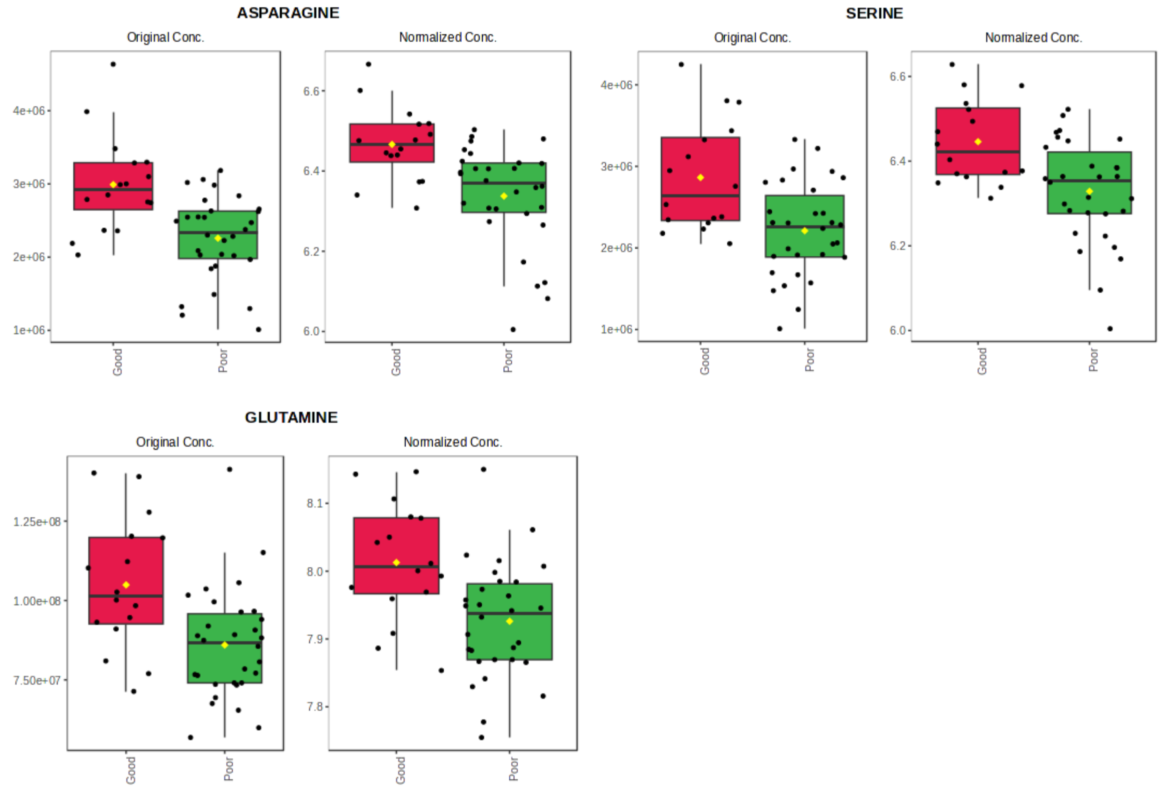

B

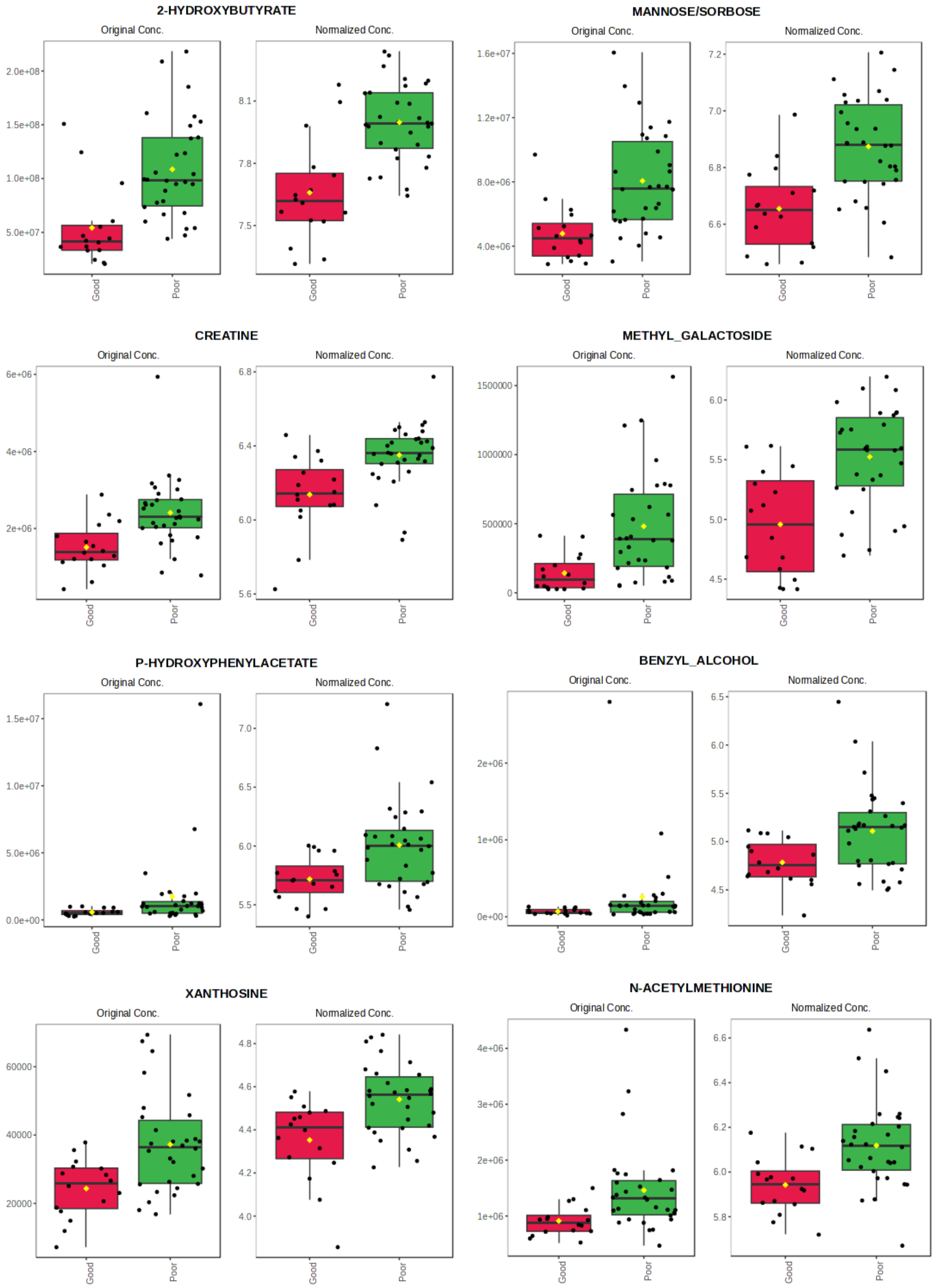
2-HYDROXYBUTYRATE
Original Conc.
Normalized Conc.
MANNOSE/SORBOSE
Original Conc.
Normalized Conc.
CREATINE
Original Conc.
Normalized Conc.
METHYL_GALACTOSIDE
Original Conc.
Normalized Conc.
P-HYDROXYPHENYLACETATE
Original Conc.
Normalized Conc.
BENZYL_ALCOHOL
Original Conc.
Normalized Conc.
XANTHOSINE
Original Conc.
Normalized Conc.
N-ACETYLMETHIONINE
Original Conc.
Normalized Conc.
Good
Poor

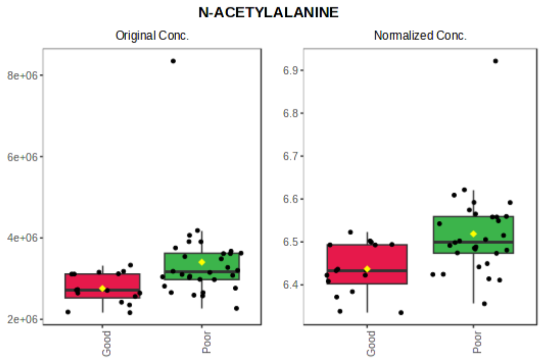
N-ACETYLALANINE
Original Conc.
Normalized Conc.
Good
Poor

C

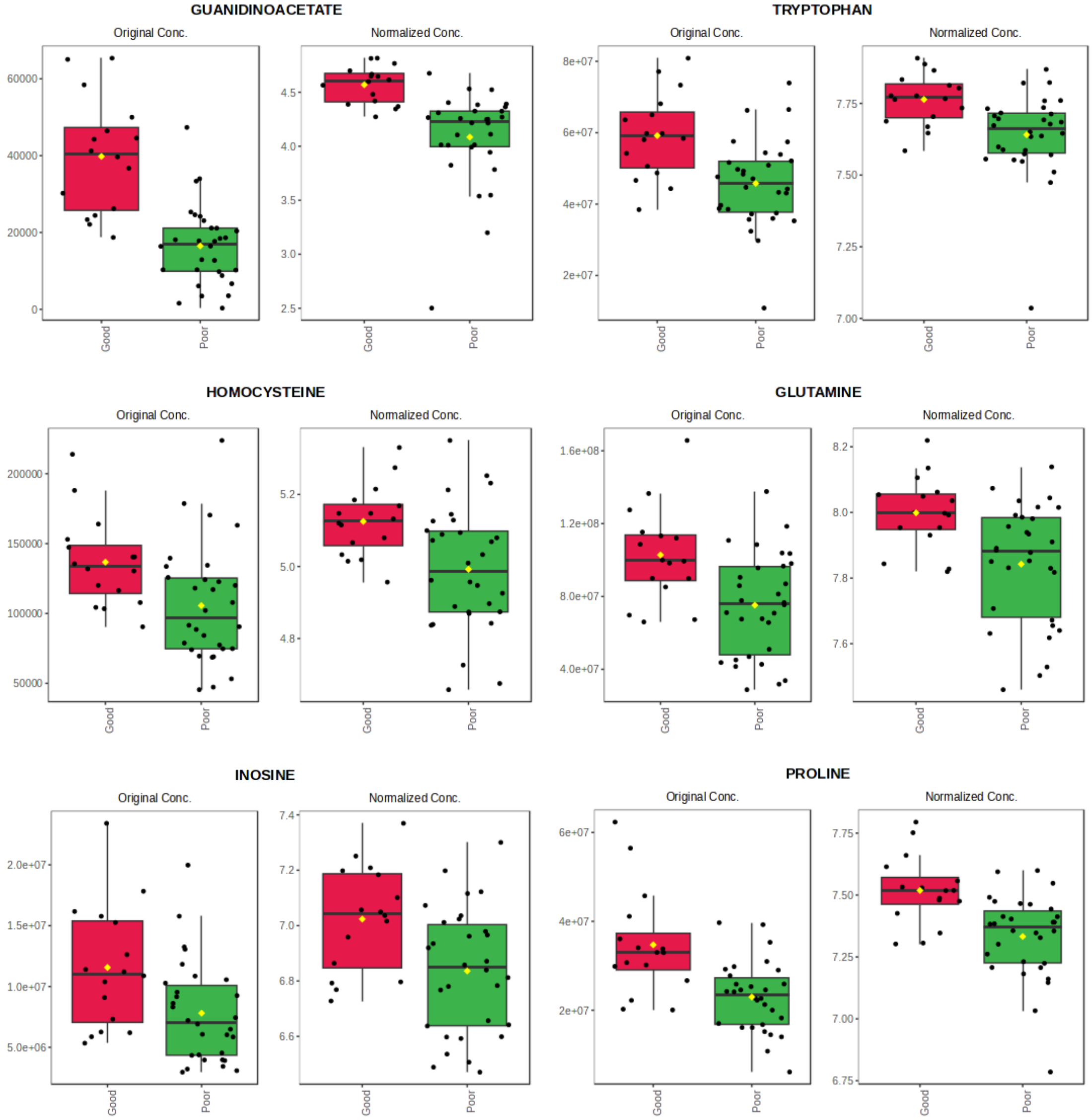
GUANIDINOACETATE
TRYPTOPHAN
HOMOCYSTEINE
GLUTAMINE
INOSINE
PROLINE
Original Conc.
Normalized Conc.
Good
Poor

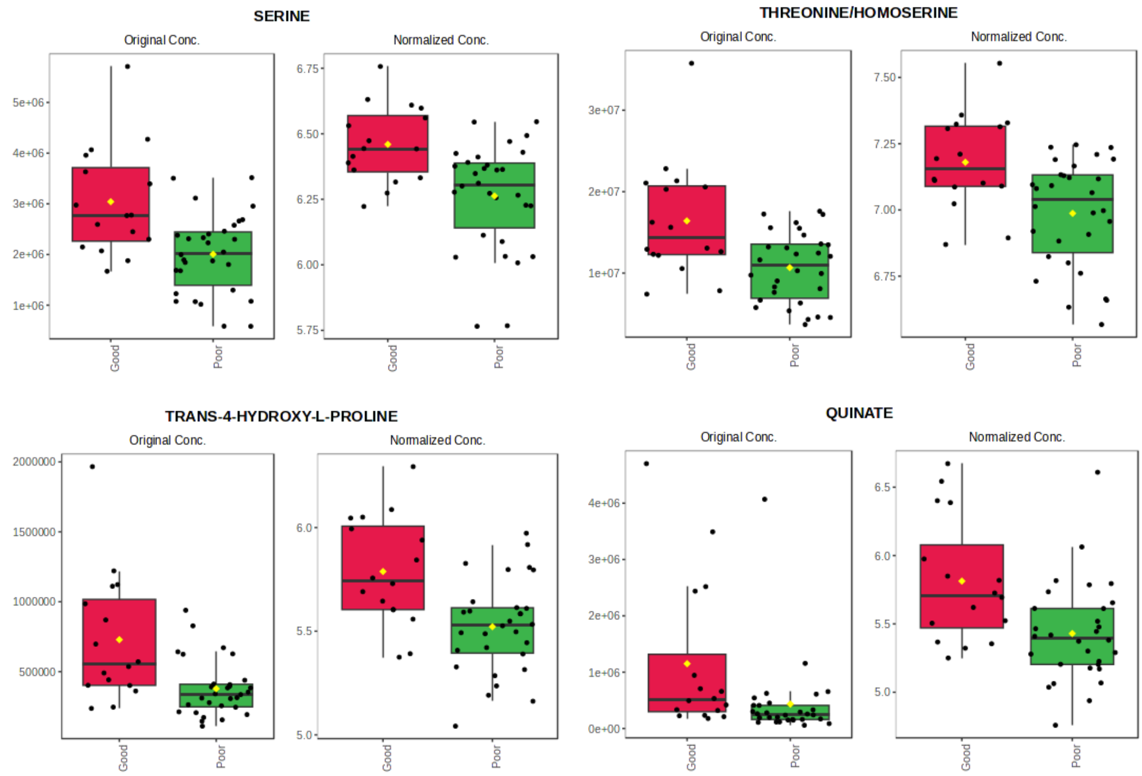

D

Figure S2. Comparison of differential metabolite intensities between the good and poor outcome groups on D1 (A) upregulated, (B) downregulated and D7 (C) upregulated, (D) downregulated.

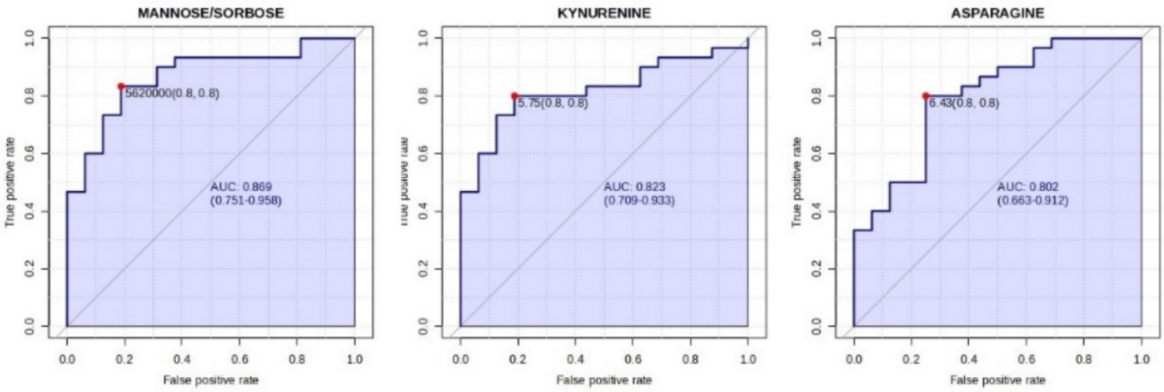

A

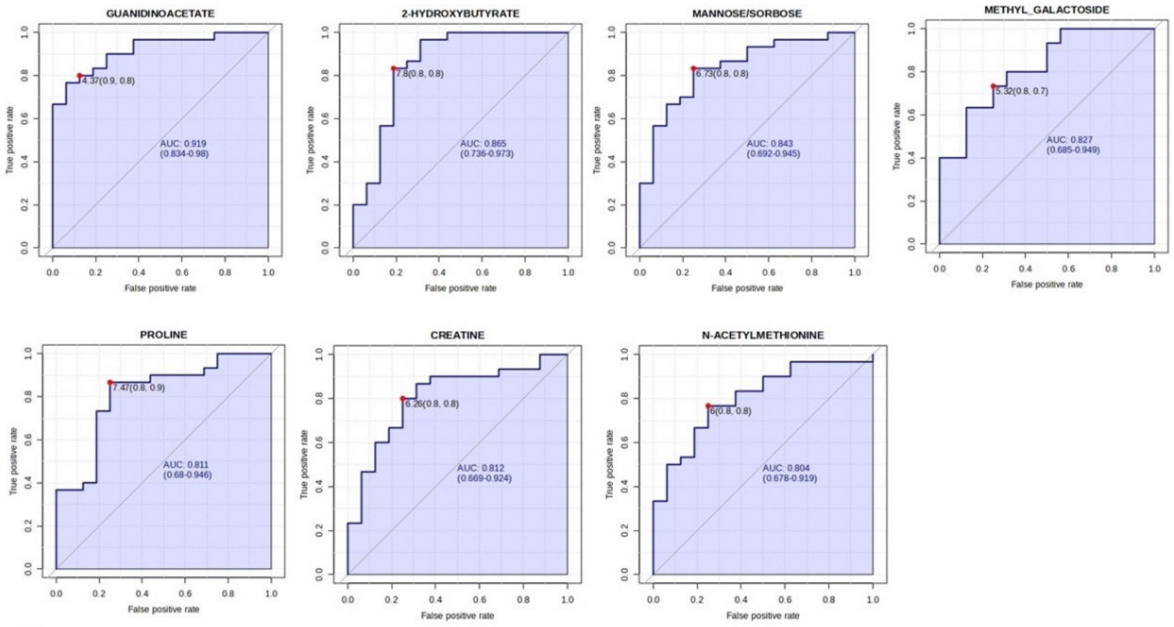

B

Figure S3. Differential metabolite ROC curves and cut-off values. (A) D1, (B) D7.
